# Manipulation of iron status on cerebral blood flow at high altitude in lowlanders and adapted highlanders

**DOI:** 10.1177/0271678X231152734

**Published:** 2023-03-08

**Authors:** Alexander Patrician, Christopher Willie, Ryan L Hoiland, Christopher Gasho, Prajan Subedi, James D Anholm, Michael M Tymko, Philip N Ainslie

**Affiliations:** 1 Centre for Heart, Lung, & Vascular Health, School of Health and Exercise Sciences, University of British Columbia – Okanagan, Kelowna, BC, Canada; 2 Department of Anesthesiology, Pharmacology and Therapeutics, Faculty of Medicine, Vancouver General Hospital, University of British Columbia, Vancouver, BC, Canada; 3 Department of Cellular and Physiological Sciences, Faculty of Medicine, University of British Columbia, Vancouver, BC, Canada; 4 International Collaboration on Repair Discoveries (ICORD), University of British Columbia, Vancouver, British Columbia, Canada; 5 Pulmonary/Critical Care Section, VA Loma Linda Healthcare System and Department of Medicine, Loma Linda University, Loma Linda, CA, USA

**Keywords:** Iron, hypoxia, cerebral blood flow, high altitude, high-altitude residents

## Abstract

Cerebral blood flow (CBF) increases during hypoxia to counteract the reduction in arterial oxygen content. The onset of tissue hypoxemia coincides with the stabilization of hypoxia-inducible factor (HIF) and transcription of downstream HIF-mediated processes. It has yet to be determined, whether HIF down- or upregulation can modulate hypoxic vasodilation of the cerebral vasculature. Therefore, we examined whether: 1) CBF would increase with iron depletion (via chelation) and decrease with repletion (via iron infusion) at high-altitude, and 2) explore whether genotypic advantages of highlanders extend to HIF-mediated regulation of CBF. In a double-blinded and block-randomized design, CBF was assessed in 82 healthy participants (38 lowlanders, 20 Sherpas and 24 Andeans), before and after the infusion of either: iron(III)-hydroxide sucrose, desferrioxamine or saline. Across both lowlanders and highlanders, baseline iron levels contributed to the variability in cerebral hypoxic reactivity at high altitude (R^2^ = 0.174, P < 0.001). At 5,050 m, CBF in lowlanders and Sherpa were unaltered by desferrioxamine or iron. At 4,300 m, iron infusion led to 4 ± 10% reduction in CBF (main effect of time p = 0.043) in lowlanders and Andeans. Iron status may provide a novel, albeit subtle, influence on CBF that is potentially dependent on the severity and length-of-stay at high altitude.

## Introduction

The brain is highly oxygen (O_2_) dependent and, as such, the related cerebral blood flow (CBF) responses to hypobaric hypoxia have been well described.^1–4^ For example, upon exposure to hypoxia, CBF increases in order to compensate for the initial reductions in arterial O_2_ content in order to maintain cerebral O_2_ delivery.^5,6^ Eventually, the acute rise in CBF is attenuated, coinciding with an increase in arterial O_2_ content via erythropoiesis, ventilatory acclimatization and compensation of the initial respiratory alkalosis.^2,7,8^

In hypoxia, the hypoxia-inducible factor (HIF) family – the key cellular O_2_ sensor^9,10^ – binds to hypoxia-responsive elements in gene promoters to up-regulate expression of >100 genes to coordinate increased O_2_ supply to hypoxic tissue. While HIF-1α expression within the human brain has not been quantified during hypoxia, data from rodent models shows that cortical HIF-1α expression during hypoxia follows a similar trajectory to the CBF responses – i.e., HIF-1α peak expression occurring with 6–12 h of exposure to hypoxia, which is halved by day 7, and normalized within ∼3 weeks.^11^ The potential for HIF expression to influence cerebrovascular function also stems from murine models, that have shown that within 4-hours of exposure to extreme hypoxia, there is an increase in downstream products of HIF-1α [e.g. vascular endothelial growth factor, erythropoietin (EPO)] in the brain, that increase cerebral microvascular density and hematocrit (Hct) within 3 weeks.^12^ Similarly, inactivation of prolyl-hydroxylase [the site whereby iron acts to influence the stability of HIF]^13^ leads to HIF expression, neurovascular angiogenesis and pericyte proliferation in mice.^14^ Finally, cerebral astrocytes (but not cerebral neurons) exposed to extreme hypoxia and desferrioxamine (DFO; iron chelator) showed an increase in EPO expression via HIF-2α up-regulation.^15^ In humans, it is reasonable that the increase in CBF that occurs to counteract the reduction in arterial O_2_ content during hypoxia, also coincides with HIF stabilization. However, it has yet to be determined whether acute HIF down- or upregulation can acutely modulate hypoxic vasodilation of the cerebral vasculature.

Iron and iron-chelation are typically utilized to down- and up-regulate HIF expression – due to iron’s constituent role in HIF stabilization, via prolyl hydroxylase activity^13^ – which have repeatedly demonstrated notable implications in pulmonary,^16–24^ and peripheral vascular regulation.^25^ However, limited data exists with respect to the cerebral vasculature. As far as the authors are aware, the only human study to date is via the assessment of intracranial blood velocity (i.e. middle cerebral artery; MCA) following iron chelation [via DFO – an up-regulator of HIF expression^26^] by Sorond and colleagues.^27,28^ While the authors conclude that DFO infusion elevated MCA velocity, compared to saline placebo, the DFO condition appeared mostly comparable to baseline in both studies. For example, MCA velocity increased by ∼1 cm · s^−1^ (level of significance not provided) from pre- to 4 hrs post-DFO in older adults^27^ and by 2 cm · s^−1^ (p > 0.05) and 4 cm · s^−1^ (p < 0.05) at 3 hrs post-DFO in young and older adults, respectively.^28^ These extremely small changes in MCA velocity are unlikely to be of physiological significance – especially if there is constriction/dilation of the MCA.^29^ Studies of volumetric blood flow to the brain during iron manipulation (via iron and chelator infusion) have not been performed during conditions where the HIF pathways are up-regulated (i.e., hypoxia).

Tibetan Sherpa have been reported to present with a unique phenotypic adaptation to high altitude characterized by a blunted ventilatory response to hypoxia,^30^ a more efficient plasma volume-hemoglobin ratio to aid exercise capacity,^31^ a reduced prevalence of excessive erythrocytosis,^32,33^ exhibit less pronounced pulmonary hypertension, and higher lung diffusing capacity [reviewed in:^34^]. Cerebral O_2_ delivery is also lower in Sherpa – and thus, potentially suggestive that Sherpa experience less deleterious cerebral consequences to hypoxia compared to lowlanders at high altitude.^3,35^ Healthy high-altitude adapted Andeans demonstrate numerous attributes that enhance their hypoxic tolerance compared to lowlanders including: elevated birth weights, increased exhaled nitric oxide (NO) concentrations, larger lungs, improved aerobic capacity and genotypic adaptations,^36–40^ and display preserved endothelial function at high altitude.^41^ While both Sherpa and Andean highlanders display adaptive characteristics to hypoxia, both also display positive selection for HIF pathway candidate genes.^42,43^ It seems plausible, therefore, that iron manipulation may also differentially impact cerebrovascular function in healthy Andeans and Sherpas who have many naturally selective traits to high altitude.

To explore these possibilities, we examined the hypothesis that CBF would increase in response to acute iron depletion (i.e. increasing HIF activity) and decrease with repletion (i.e. decreasing HIF activity). Furthermore, we hypothesized that during exaggerated hypoxia at high altitude, the increase in CBF would be amplified by chelation, and attenuated by iron infusion. Finally, by assessing high altitude populations with ancestral adaptation to hypoxic exposure, we sought to explore whether the genotypic advantages of Andeans and Sherpas, related to iron metabolism, would manifest through the modulation of CBF.

## Materials and methods

### Ethical approval

All experimental procedures were approved by the University of British Columbia Research Ethics Board (H16-01028, H17-02687 and H18-01404), the Nepal Health Research Council (no. 586), the Universidad Peruana Cayetano Heredia Comité de Ética (no. 101686), and conformed to the Declaration of Helsinki, except for registration in a database. All participants received both written and oral information about the study and provided written informed consent. All highlander (Sherpa and Andean) participants read an in-depth translated study information form and had the study explained to them in their local language, and gave written informed consent prior to participating.

### Experimental design

Data collected during this study have previously been published as part of an investigation that focused exclusively on the pulmonary and peripheral vascular responses to iron manipulation.^16,26,44^ Thus, although the present study adopted an identical experimental design, it constitutes an entirely separate research question complemented by *de novo* experimental measures constrained to the cerebral vasculature.

### Participants

A total of 82 volunteers participated in the study, which was conducted across two high altitude research expeditions.^45,46^ Participants consisted of 38 lowlanders (30 ± 8 years, 176 ± 7 cm, 73 ± 9 kg, 23 ± 2 kg · m^−2^), 20 Sherpa (25 ± 7 years, 169 ± 6 cm, 64 ± 11 kg, 22 ± 3 kg · m^−2^) and 24 Andeans (29 ± 11 years, 162 ± 5 cm, 63 ± 7 kg, 24 ± 3 kg · m^−2^). Andeans were not considered to suffer from chronic mountain sickness [CMS;^47^], as they did not exhibit excessive erythrocytosis (hemoglobin ≤19 g · dl^−1^ for females and ≤21 g · dl^−1^ for males) and had a Qinghai CMS questionnaire score of 0.5 ± 0.8.

#### Study 1 – Lowlanders and Sherpa at 5,050 m

**Figure 1.**
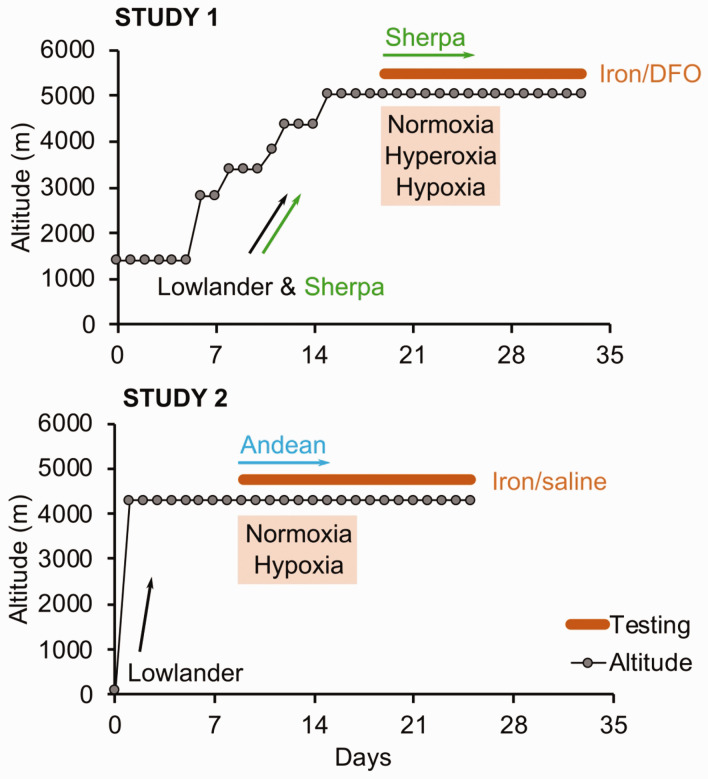
Summary of experimental protocol of each study. In Study 1, lowlanders and Sherpa hiked to 5050 m over 9–10 days. Prior to flying to Lukla: the ascending Sherpa group had already descended and been in Kathmandu for 5–15 days (median: 7 days); lowlanders had been in Kathmandu (1400 m) for 3–9 days (median: 6 days). Additional Sherpa were recruited at high altitude – typically ascending from 3800–4200 m in 1–2 days, and were tested 1–2 days following arrival to 5050 m. In Study 2, lowlanders were driven over 8 hours from Lima to 4300 m, Cerro de Pasco (where all Andeans were residents). Experimental P_I_O_2_ conditions are included, that were repeated pre- and post-infusion.

At 5,050 m in the Nepalese Himalaya (EV-K2-CNR Pyramid Research laboratory), 7 male lowlanders and 8 male Sherpa received an infusion of DFO (desferrioxamine; 7 mg/kg/hour over 4 hr) and 9 male lowlanders and 12 male Sherpa received an infusion of iron [iron (iii)-hydroxide sucrose; 200 mg over 30 min followed by 3.5 hours of slow-drip saline (0.9% NaCl); the total infusion time for iron was 4 hr to mirror the time-course of the DFO infusion]. Lowlanders receiving DFO and iron were tested after 13 ± 3 days and 12 ± 4 days, respectively at 5,050 m (Figure 1). There were 8 Sherpa who ascended from Kathmandu and tested following 7 ± 3 days at 5,050 m, and then 12 Sherpa who ascended from 3,800–4,200 m, and were tested 1–2 days following arrival to 5,050 m.

#### Study 2 – Lowlanders and Andeans at 4300 m

At 4,300 m in the Peruvian Andes (Cerro de Pasco – resident altitude for Andeans), 11 lowlanders (2 female) and 12 Andeans (1 female) received an infusion of saline (250 ml of 0.9% NaCl saline) and 11 lowlanders (4 female) and 12 Andeans (2 female) received an infusion iron sucrose [iron (III)-hydroxide sucrose; 200 mg in 250 ml 0.9% NaCl saline] over 30 min. Lowlanders receiving saline and iron were tested after 9 ± 5 and 6 ± 3 days, respectively (Figure 1).

#### Infusion randomization

For Studies 1 & 2, block-randomization (i.e. randomization performed in stages, across the expedition) of participants was performed for three primary purposes: 1) it streamlined data collection; 2) optimized coordination with other ongoing studies (i.e. avoid any potential confounding effects of manipulated iron levels); and 3) ensured that lowlanders allocated to iron and saline or DFO conditions were appropriately weighted, in terms of the number of days at altitude, to limit any potentially confounding influence of hypobaric exposure on iron stores. Analysis was conducted on coded data/files.

### Changes in P_I_O_2_

Measures of CBF (see *experimental measures* below), blood pressure, peripheral O_2_ saturation via pulse oximetry (SpO_2_) and ventilation (via Wright spirometer) were collected during resting supine breathing of: 1) ambient air (P_I_O_2_ of 87 mmHg at 5,050 m and P_I_O_2_ of 96 mmHg at 4,300 m), and 2) exaggerated hypoxia (P_I_O_2_ of 67 mmHg at 5,050 m and P_I_O_2_ of 73 mmHg at 4,300 m; simulating an additional elevation gain of ∼2000 m each), both before and after the infusion (see *Participants* above). The partial pressure of end-tidal CO_2_ (P_ET_CO_2_) was assessed using capnography (EMMA, Masimo); however, whilst Douglas bag breathing (i.e., exaggerated hypoxia), P_ET_CO_2_ was not collected due to the increased physiological deadspace associated with our respiratory apparatus. Consequently, P_ET_CO_2_ during exaggerated hypoxia was calculated, based on the change in alveolar ventilation (see *Data Analysis* below).

### Experimental measures

*Cerebral blood flow:* Internal carotid artery (ICA) and vertebral artery (VA) diameter with synchronous measurements of blood velocity were performed using a 10-MHz multifrequency linear array probe with high-resolution duplex ultrasound (Terason t3200 and Terason uSmart 3300, Teratech). The ICA was measured at least 2 cm from the carotid bifurcation, whilst avoiding turbulent or retrograde flow. The VA was measured approximately at the transverse process of C4 and the subclavian artery. Each ICA and VA measures were conducted at the same location within each subject. Images of blood vessel diameter and blood velocity were recorded as video files, which were analyzed offline using an automated edge-detection software [FMD/BloodFlow Software version 5.1, Reed C, Australia;^48^]. All data are based on imaging of >15 cardiac cycles, with stable and repeated angle of insonation. Volumetric blood flow (Q) was quantified using the following equation:

$$Q_{\mathrm{ICA}}\text{ or }Q_{VA}=\left\{\left(\mathrm{peak}\;\mathrm{envelope}\;\mathrm{velocity}/2\right)\cdot \left[{(\pi \cdot \mathrm{diameter}/2)}^{2}\right]\right\}\times 60$$

Global CBF (gCBF) was estimated as twice the sum of unilateral ICA and VA flows. Cerebrovascular conductance (CVC) was estimated using MAP:

CVC=Q/MAP

gCBF reactivity was estimated as ΔgCBF/ΔSpO_2_. Automated blood pressure was collected in duplicate using brachial oscillometry. The absolute change in blood flow (ΔQ) from room air to exaggerated hypoxia was used to assess hypoxic reactivity of a particular vessel (e.g. ΔQ_VA_) and the bulk flow to the brain (i.e. ΔgCBF).

#### Blood sampling

Venous blood samples were separated by microcentrifugation, with serum samples frozen in liquid nitrogen at −196 °C for analysis. Serum iron was analysed according to clinical laboratory standards (Samyak Diagnostic Pvt. Ltd., Kathmandu, Nepal and Medlab Clinical Laboratories, Lima, Peru). Hemoglobin concentration ([Hb]) and hematocrit (Hct) were obtained from whole venous blood sample and analyzed immediately (ABL90 FLEX, Radiometer and microcentrifugation).

### Data analysis

P_ET_CO_2_ during poikilocapnic hypoxia was calculated using a mean slope of P_ET_CO_2_ [derived from 15 min of poikilocapnic hypoxia in 22 healthy individuals^49^] per change in V_E_ from room air breathing to hypoxia:

$$\left[\left({V_{E}}_{\mathrm{hypox}}-{V_{E}}_{\mathrm{normox}}\right)\times (-0.4)\right]+{P_{ET}{CO}_{2}}_{\mathrm{normox}}$$

### Statistical analysis

Data were analyzed using a linear mixed effects model with a compound symmetry repeated measure co-variance structure (SPSS v24, IBM Statistics). The fixed factors for the model were ancestry and time (i.e., pre to post infusion) – with the latter being a repeated factor with a compound symmetry repeated covariance structure. When a significant interaction effect (e.g., ancestry × time) was detected, Bonferroni adjusted post-hoc tests were utilized to test pairwise comparisons. Pre- and post-infusion iron markers were assessed using paired samples t-test. Correlations were assessed using Pearson correlation. A one-way ANOVA was used to assess the absolute change in serum iron across groups. Shapiro-Wilk normality testing confirmed primary outcome measure data (gCBF, serum iron, Hb, Hct, MAP, SpO_2_) were normally distributed. All results are reported as mean ±SD and significance was set at P < 0.05.

## Results

### Baseline iron status on global cerebral blood flow

**Figure 2.**
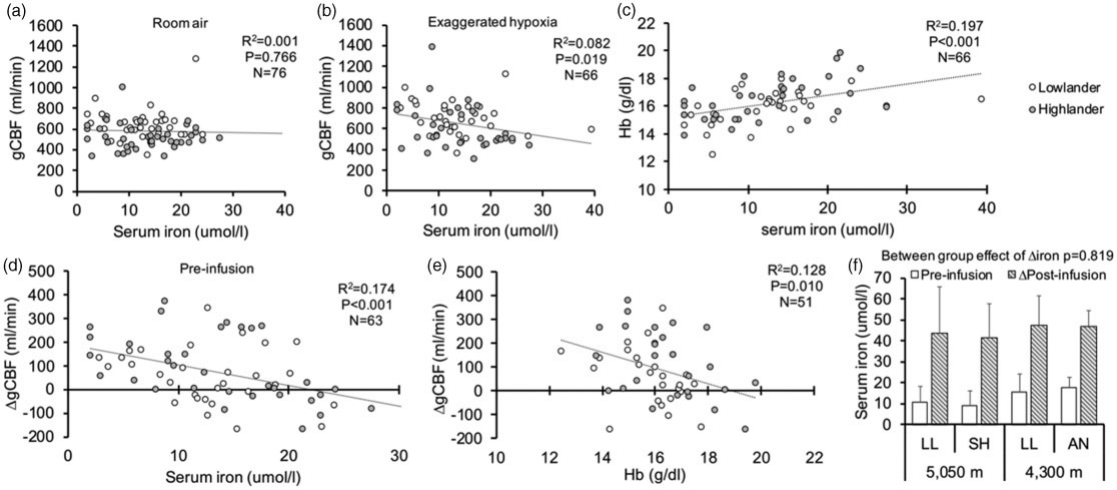
Influence of baseline iron status on gCBF in pooled lowlanders and pooled highlanders at high altitude. Panels a and b illustrate the relationship between gCBF during room air breathing and exaggerated hypoxia. Panel d indicates that the change in hypoxic reactivity of gCBF is greater in those with lower iron levels, compared to those with more elevated iron levels. Panel C and E highlight the relationship between baseline iron and [Hb], with hypoxic reactivity of gCBF, respectively. Panel f highlights serum iron levels in each group [lowlanders (LL), Andeans (AN) and Sherpas (SH) at their respective altitudes] at baseline (i.e. pre-infusion), and the absolute increase in serum iron following iron infusion (i.e. Δpost-infusion).

When data from lowlanders and highlanders were pooled across both expeditions, baseline iron status was not associated with gCBF during room air breathing (R^2^ = 0.001, P = 0.766; Figure 2(a)); however, during exaggerated hypoxia, iron status was significantly correlated with gCBF (R^2^ = 0.082, P = 0.019; Figure 2(b)). Consequently, as illustrated in Figure 2(d), variations in baseline and physiologically normal iron levels appears to influence hypoxic reactivity of gCBF (R^2^ = 0.174, P < 0.001). Hb was also correlated with serum iron (R^2^ = 0.197; P < 0.001; Figure 2(c)); therefore, Hb and Hct were both associated with ΔgCBF (R^2^ = 0.128, P = 0.010 and R^2^ = 0.150, P = 0.003; Figure 2(e)).

### Between study infusion comparability

The effectiveness of iron infusion to increase serum iron was comparable across lowlanders and Andeans at 4,300 m and lowlanders and Sherpa 5,050 m (P = 0.819; Figure 2(f)). Saline infusion did not alter serum iron in either lowlanders or Andeans (main effect of time P = 0.759), whereas DFO decreased serum iron by ∼60%, to nearly undetectable levels of 2.4 ± 0.9 µmol · l^−1^ in lowlanders and 2 ± 0 µmol · l^−1^ in Sherpa (main effect of time P < 0.001).

### Study 1: DFO and iron at 5,050 m

In both lowlanders and Sherpa, V_E_, P_ET_CO_2_, SpO_2_ and MAP were unaltered by either DFO or iron. Ultimately, there was no change in Q_ICA_, Q_VA_, gCBF or CVC following either DFO or iron, in lowlanders and Sherpa (Table 1). Moreover, and in contrast to our hypotheses, during exaggerated hypoxia (P_I_O_2_ =67 mmHg), DFO did not increase gCBF and iron did not lower gCBF in either lowlanders or Sherpa (Table 2). When normalized to the change in SpO_2_, the reactivity of gCBF (i.e., ΔgCBF/ΔSpO_2_) during hypoxia was also not altered by iron or DFO manipulation (Table 2).

**Table 1.** During room air breathing (P_I_O_2_ = 87 mmHg) at pre- and post-infusion at 5,050 m in lowlanders and Sherpa.

		Pre-DFO	Post-DFO	Ancestry	Time	Inter		Pre-iron	Post-iron	Ancestry	Time	Inter
Q_ICA_(ml · min^−1^)	Lowlander	245 ± 59	276 ± 46 [6]	0.270	0.326	0.179		259 ± 59 [7]	264 ± 59 [8]	**0.046**	0.909	0.441
	Sherpa	216 ± 82 [7]	218 ± 69 [6]					209 ± 53	202 ± 64			
Q_VA_(ml · min^−1^)	Lowlander	74 ± 13	82 ± 23 [6]	0.313	0.880	0.130		63 ± 27	62 ± 26	0.261	0.233	0.393
	Sherpa	72 ± 29 [7]	64 ± 19					52 ± 24 [11]	51 ± 22 [9]			
gCBF(ml · min^−1^)	Lowlander	639 ± 135	718 ± 125 [6]	0.280	0.433	0.148		655 ± 99 [7]	652 ± 111 [8]	**0.010**	0.929	0.592
	Sherpa	577 ± 214 [7]	568 ± 175 [6]					512 ± 125 [11]	515 ± 110 [9]			
CVC(ml · min^−1^mmHg^−1^)	Lowlander	7.23 ± 1.65	8.11 ± 1.52 [6]	0.516	0.136	0.668		6.78 ± 1.2 [7]	6.74 ± 1.39 [8]	0.106	0.785	0.585
	Sherpa	6.64 ± 2.6 [7]	7.12 ± 2.25 [6]					5.87 ± 1.33 [11]	5.81 ± 1.25 [9]			
Systolic(mmHg)	Lowlander	120 ± 4	122 ± 8	0.976	0.190	**0.010**		130 ± 7	129 ± 9	**0.018**	0.974	0.685
	Sherpa	124 ± 11	118 ± 11*					117 ± 11	118 ± 16			
Diastolic(mmHg)	Lowlander	74 ± 10	74 ± 8	0.549	0.452	0.311		82 ± 9	83 ± 7	**0.019**	0.811	0.784
	Sherpa	73 ± 9	69 ± 14					73 ± 9	73 ± 12			
MAP(mmHg)	Lowlander	89 ± 7	90 ± 7	0.644	0.277	0.078		98 ± 8	98 ± 7	**0.014**	0.849	0.956
	Sherpa	90 ± 9	85 ± 12					88 ± 9	88 ± 12			
V_E_(l · min^−1^)	Lowlander	10.1 ± 1.4	12.1 ± 2.6	**0.006**	0.091	0.705		11.7 ± 1.9	12.9 ± 2.1	0.821	**0.048**	0.905
	Sherpa	14.8 ± 3.5	16 ± 3.8					11.4 ± 3.5	12.7 ± 3.5			
P_ET_CO_2_(mmHg)	Lowlander	28 ± 2	29 ± 3	**0.027**	0.626	0.788		28 ± 2	29 ± 3	0.248	0.874	0.447
	Sherpa	31 ± 2	32 ± 3					30 ± 3	30 ± 3			
SpO_2_(%)	Lowlander	87 ± 2	87 ± 2	0.482	0.542	0.838		88 ± 3	86 ± 2	0.374	0.321	0.059
	Sherpa	86 ± 3	86 ± 3					86 ± 4	86 ± 3			
Heart rate(bpm)	Lowlander	60 ± 7	65 ± 10	**0.030**	0.275	0.265		66 ± 12	61 ± 9	0.024	0.158	0.103
	Sherpa	72 ± 9	72 ± 9					74 ± 9	74 ± 12			
Hct (%)	Lowlander	47 ± 3	45 ± 3 [6]	0.678	0.184	0.820		45 ± 3	46 ± 3	0.452	0.129	0.087
	Sherpa	46 ± 3	45 ± 3					47 ± 4	46 ± 3			
Hb (g dl^−1^)	Lowlander	15.8 ± 0.9	15.3 ± 1 [6]	0.870	0.071	0.895		15.5 ± 1	15.5 ± 1.1	0.508	0.100	0.100
	Sherpa	15.7 ± 0.9	15.4 ± 1					16.0 ± 1.1	15.5 ± 1			
Serum iron(µmol · l^−1^)	Lowlander	11.7 ± 7.5	2.4 ± 0.9	0.206	**<0.001**	0.243		10.8 ± 7.5	53.8 ± 22.6	0.606	**<0.001**	0.936
	Sherpa	7.6 ± 4.7	2 ± 0					8.9 ± 7.1	51.2 ± 15.7 [11]			

Mean ± SD in 7 lowlanders and 8 Sherpa receiving DFO and 9 lowlanders and 12 Sherpa receiving iron, unless otherwise noted [n]. Q: blood flow; gCBF: global cerebral blood flow; CVC: cerebrovascular conductance; ICA: internal carotid artery; Hb: hemoglobin; Hct: hematocrit; MAP: mean arterial pressure; P_ET_CO_2:_ partial pressure of end-tidal carbon dioxide; SpO_2_: peripheral arterial oxygen saturation; V_E_: ventilation; VA: vertebral artery. Significant main effects are bolded. *P < 0.05 from baseline.

**Table 2.** Absolute change from room air (P_I_O_2_ = 87mmHg) to hypoxia (P_I_O_2_ = 67mmHg) in lowlanders and Sherpa.

		Pre-DFO	Post-DFO	Ancestry	Time	Inter	Pre-iron	Post-iron	Ancestry	Time	Inter
ΔQ_ICA_ (ml · min^−1^)	Lowlander	47 ± 26 [6]	47 ± 35 [6]	0.651	0.550	0.559	7 ± 59 [7]	63 ± 98 [8]	0.384	0.212	0.095
	Sherpa	69 ± 61 [7]	49 ± 79 [6]				57 ± 36 [8]	52 ± 55 [8]			
ΔQ_VA_ (ml · min^−1^)	Lowlander	15 ± 16 [5]	30 ± 18 [6]	0.778	0.823	**0.047**	20 ± 23 [8]	26 ± 24	0.738	0.816	0.123
	Sherpa	27 ± 29 [6]	13 ± 15 [7]				20 ± 17 [10]	16 ± 23 [9]			
ΔgCBF (ml · min^−1^)	Lowlander	138 ± 62 [5]	154 ± 91 [6]	0.608	0.309	0.179	73 ± 169 [6]	174 ± 222 [8]	0.777	0.502	0.085
	Sherpa	226 ± 141 [6]	133 ± 184 [5]				157 ± 93 [7]	124 ± 85 [5]			
ΔCVC (ml · min^−1^mmHg^−1^)	Lowlander	0.7 ± 0.69 [5]	1.91 ± 0.74 [6]	0.436	0.839	**0.045**	0.54 ± 1.65 [6]	1.55 ± 2.32 [8]	0.402	0.886	**0.030**
	Sherpa	2.7 ± 1.94 [6]	1.39 ± 2.61 [5]				2.12 ± 1.53 [7]	0.96 ± 0.84 [5]			
ΔgCBF/ΔSpO_2_ (ml · min^−1^%^−1^)	Lowlander	−12.4 ± 7.2 [5]	−7 ± 4.2 [4]	0.895	0.071	0.575	−0.1 ± 25.7 [6]	−16 ± 24.9 [7]	0.540	0.165	0.241
	Sherpa	−11.3 ± 7.1 [6]	−7.4 ± 8.4 [5]				−0.6 ± 18.3 [7]	−6.1 ± 3.3 [4]			
ΔMAP(mmHg)	Lowlander	8 ± 5 [6]	−2 ± 10 [6]*	0.435	0.181	**0.002**	1 ± 6	2 ± 5	0.862	0.091	0.156
	Sherpa	−2 ± 6†	3 ± 8				−2 ± 8	6 ± 6			
ΔP_ET_CO_2_ (mmHg)	Lowlander	−3 ± 1 [6]	−2 ± 2 [6]	0.260	0.973	0.213	−4 ± 3	−2 ± 1	0.176	**0.016**	0.673
	Sherpa	−1 ± 2	−2 ± 3				−2 ± 2	−1 ± 2			
ΔV_E_ (l · min^−1^)	Lowlander	7 ± 3 [6]	5 ± 4 [6]	0.260	0.973	0.213	9 ± 8	5 ± 2	0.176	**0.016**	0.673
	Sherpa	2 ± 6	5 ± 7				6 ± 5	3 ± 5			
ΔSpO_2_ (%)	Lowlander	−11 ± 3 [6]	−16 ± 12 [6]	0.137	0.634	0.441	−13 ± 5	−12 ± 8	**0.049**	0.838	0.706
	Sherpa	−19 ± 6	−18 ± 10				−18 ± 10	−19 ± 9 [11]			

Mean ± SD in 7 lowlanders and 8 Sherpa receiving DFO and 9 lowlanders and 12 Sherpa receiving iron, unless otherwise noted [n]. P_ET_CO_2_ during hypoxia was predicted. Δ: absolute change; Q: blood flow; CVC: cerebrovascular conductance; gCBF: global cerebral blood flow; ICA: internal carotid artery; MAP: mean arterial pressure; P_ET_CO_2_: partial pressure of end-tidal carbon dioxide; SpO_2_: peripheral arterial oxygen saturation; V_E_: ventilation; VA: vertebral artery. Significant main effects are bolded. *P < 0.05 from baseline, †P < 0.05 from lowlanders.

### Study 2: Saline and iron at 4,300 m

In both lowlanders and Andeans, V_E_, P_ET_CO_2_, SpO_2_ and MAP were unaltered by saline and iron infusions (Table 3). While saline infusion did not alter gCBF, iron infusion led to a 4 ± 10% reduction in gCBF (main effect of time p = 0.043) and a 7 ± 13% decrease in CVC (main effect of time p = 0.011) in both lowlanders and Andeans (Table 3). During exaggerated hypoxia (P_I_O_2_ = 73 mmHg), however, neither saline or iron, altered the rise in gCBF. The reactivity of gCBF during hypoxia (i.e., ΔgCBF/ΔSpO_2_) was attenuated following iron infusion (main effect of time P = 0.019; Table 4). In only lowlanders at 4,300 m, the change in VA blood flow from room air to hypoxia (i.e. ΔQ_VA_) increased from 4 ± 13 ml · min^−1^ pre-iron, to 21 ± 19 ml · min^−1^ post-iron infusion (p = 0.023).

**Table 3.** During room air breathing (P_I_O2 = 96 mmHg) at pre- and post-infusion at 4,300 m in lowlanders and healthy Andeans.

		Pre-saline	Post-saline	Ancestry	Time	Inter	Pre-iron	Post-iron	Ancestry	Time	Inter
Q_ICA_ (ml · min^−1^)	Lowlander	260 ± 122	265 ± 119	0.148	0.905	0.560	257 ± 53 [10]	234 ± 59	**0.028**	**0.043**	0.922
	Andean	211 ± 45	197 ± 51 [11]				202 ± 34	188 ± 31			
Q_VA_ (ml · min^−1^)	Lowlander	57 ± 27	63 ± 34	0.408	0.202	0.408	74 ± 21	76 ± 24 [10]	**0.014**	0.606	0.939
	Andean	51 ± 19	52 ± 19				53 ± 15	52 ± 18			
gCBF (ml · min^−1^)	Lowlander	635 ± 240	657 ± 221	0.090	0.761	0.389	661 ± 86 [10]	611 ± 100 [10]	**<0.001**	**0.043**	0.992
	Andean	525 ± 87	502 ± 113 [11]				511 ± 65	480 ± 54			
CVC (ml · min^−1^mmHg^−1^)	Lowlander	6.98 ± 3.06	7.27 ± 2.6	0.441	0.753	0.474	7.54 ± 0.66 [10]	6.8 ± 1.35 [10]	0.246	**0.011**	0.592
	Andean	6.51 ± 0.92	6.27 ± 1.25 [11]				6.92 ± 1.23	6.2 ± 1.22			
Systolic (mmHg)	Lowlander	123 ± 12	124 ± 12	**0.008**	0.546	0.613	120 ± 10	120 ± 10	**<0.001**	0.415	0.469
	Andean	109 ± 7	111 ± 16				103 ± 8	106 ± 11			
Diastolic (mmHg)	Lowlander	76 ± 7	75 ± 9	**0.037**	0.881	0.405	73 ± 8	76 ± 6	**0.002**	**0.011**	0.447
	Andean	66 ± 9	69 ± 13				61 ± 7	66 ± 11			
MAP (mmHg)	Lowlander	92 ± 8	91 ± 10	**0.015**	0.738	0.438	89 ± 8	91 ± 7	**<0.001**	**0.038**	0.406
	Andean	81 ± 8	83 ± 14				75 ± 6	79 ± 11			
V_E_ (l · min^−1^)	Lowlander	11 ± 2 [9]	10 ± 2 [9]	0.498	0.506	0.606	8.2 ± 1.6 [10]	7.8 ± 1.6 [9]	**<0.001**	0.811	0.313
	Andean	12 ± 4	12 ± 4				12.6 ± 2.5	13.4 ± 4.3			
P_ET_CO_2_ (mmHg)	Lowlander	30 ± 3	31 ± 3	0.357	0.756	0.653	33 ± 3 [8]	32 ± 2 [8]	0.326	0.264	0.792
	Andean	32 ± 3	32 ± 3				31 ± 3	31 ± 4			
SpO_2_ (%)	Lowlander	89 ± 3	88 ± 4	0.694	0.596	0.596	88 ± 3	88 ± 3 [10]	0.453	0.110	0.218
	Andean	88 ± 2	88 ± 2				88 ± 4	87 ± 3			
HR (bpm)	Lowlander	68 ± 11	62 ± 14	0.758	**0.027**	0.852	71 ± 14	66 ± 16	0.421	**<0.001**	0.295
	Andean	66 ± 12	61 ± 9				69 ± 9	60 ± 8			
Hct (%)	Lowlander	48 ± 4	49 ± 5	**0.009**	0.696	0.495	48 ± 3 [9]	48 ± 3 [10]	**0.011**	0.801	0.566
	Andean	54 ± 4	54 ± 6 [9]				51 ± 5 [6]	54 ± 5 [8]			
Hb (g dl^−1^)	Lowlander	16.1 ± 1.6 [9]	16.1 ± 1.7 [9]	**0.048**	0.946	0.936	16.4 ± 1.3 [8]	16.1 ± 1.3 [9]	0.280	0.715	0.180
	Andean	17.7 ± 1.9 [6]	18 ± 2 [7]				16.4 ± 1.9 [8]	17.3 ± 2.1 [11]			
Serum iron (µmol·l^−1^)	Lowlander	13.7 ± 6.3	13.4 ± 6	0.238	0.759	0.641	15.6 ± 8.4	63.2 ± 7.7 [10]	0.355	**<0.001**	0.894
	Andean	16.2 ± 5.6	17.3 ± 9.8				17.6 ± 5.1 [11]	65.7 ± 9.9			

Mean ± SD in 11 lowlanders and 12 Andeans receiving saline or iron, unless otherwise noted [n]. Q: blood flow; gCBF: global cerebral blood flow; CVC: cerebrovascular conductance; ICA: internal carotid artery; MAP: mean arterial pressure; P_ET_CO_2_: partial pressure of end-tidal carbon dioxide; SpO_2_: peripheral arterial oxygen saturation; V_E_: ventilation; VA: vertebral artery. Significant main effects are bolded.

**Table 4.** Absolute change from room air (P_I_O_2_ = 96 mmHg) to hypoxia (P_I_O_2_ = 73 mmHg) in lowlanders and healthy Andeans.

		Pre-saline	Post-saline	Ancestry	Time	Inter	Pre-iron	Post-iron	Ancestry	Time	Inter
ΔQ_ICA_ (ml · min^−1^)	Lowlander	5 ± 61	19 ± 78 [9]	0.699	0.687	0.508	19 ± 33 [10]	39 ± 30 [10]	0.717	0.299	0.589
	Andean	7 ± 43	4 ± 30 [10]				18 ± 59	21 ± 57 [11]			
ΔQ_VA_ (ml · min^−1^)	Lowlander	9 ± 12	2 ± 9 [10]	0.831	0.257	0.271	4 ± 13 [9]	21 ± 19 [9]*	0.090	0.387	**0.014**
	Andean	6 ± 11 [11]	6 ± 9 [9]				7 ± 12 [10]	−2 ± 17 [10]†			
ΔgCBF (ml · min^−1^)	Lowlander	27 ± 121	52 ± 151 [8]	0.604	0.902	0.521	35 ± 92 [8]	116 ± 87 [9]	0.572	0.267	0.221
	Andean	24 ± 103 [11]	15 ± 72 [8]				51 ± 141 [10]	39 ± 157 [9]			
ΔCVC (ml · min^−1^mmHg^−1^)	Lowlander	−0.14 ± 1.32	0.04 ± 1.4 [8]	0.835	0.905	0.630	0.1 ± 1.01 [8]	1.16 ± 1.05 [9]	0.956	0.136	0.475
	Andean	−0.07 ± 1.27 [11]	−0.03 ± 0.61 [8]				0.44 ± 1.99 [10]	0.69 ± 2.09 [9]			
ΔgCBF/ΔSpO_2_ (ml · min^−1^%^−1^)	Lowlander	0.4 ± 9.1 [11]	−10.8 ± 27 [8]	0.849	0.400	0.208	−1.5 ± 5.2 [8]	−6.8 ± 5.7 [8]	0.863	**0.019**	0.593
	Andean	−5.4 ± 12.9 [11]	−3.1 ± 9.7 [8]				−1.5 ± 8.2 [10]	−5 ± 8.7 [8]			
ΔSystolic (mmHg)	Lowlander	3 ± 12	7 ± 7	0.761	0.185	0.625	−1 ± 9	2 ± 6	0.951	0.850	0.345
	Andean	3 ± 8	5 ± 8				2 ± 11	−1 ± 6			
ΔDiastolic (mmHg)	Lowlander	6 ± 8	5 ± 6	0.461	0.701	0.919	4 ± 10	1 ± 7	0.614	**0.038**	0.661
	Andean	4 ± 9	4 ± 5				6 ± 6	1 ± 6			
ΔMAP (mmHg)	Lowlander	5 ± 7	6 ± 6	0.495	0.760	0.888	3 ± 9	1 ± 6	0.718	0.145	0.468
	Andean	4 ± 8	4 ± 5				5 ± 7	1 ± 6			
ΔP_ET_CO_2_ (mmHg)	Lowlander	−1 ± 1 [9]	−1 ± 1 [9]	0.070	0.248	0.103	−1 ± 3 [7]	−2 ± 0.4 [7]	**0.036**	0.472	0.422
	Andean	−1 ± 1	0.3 ± 1				0.1 ± 1 [11]	0.1 ± 2			
ΔV_E_ (l · min^−1^)	Lowlander	2 ± 3 [9]	3 ± 2 [9]	0.070	0.248	0.103	3 ± 5 [10]	5 ± 2 [10]	**0.015**	0.350	0.252
	Andean	1 ± 3	−1 ± 3				−0.2 ± 4 [11]	−0.2 ± 5			
ΔSpO_2_ (%)	Lowlander	−15 ± 7	−15 ± 7	0.886	0.772	0.944	−18 ± 3	−18 ± 4 [10]	0.238	0.966	0.749
	Andean	−15 ± 10	−15 ± 8				−15 ± 8	−15 ± 10			

Mean ± SD in 11 lowlanders and 12 Andeans receiving saline or iron, unless otherwise noted [n]. P_ET_CO_2_ during hypoxia was predicted. Δ: absolute change; Q: blood flow; CVC: cerebrovascular conductance; gCBF: global cerebral blood flow; ICA: internal carotid artery; MAP: mean arterial pressure; P_ET_CO_2_: partial pressure of end-tidal carbon dioxide; SpO_2_: peripheral arterial oxygen saturation; V_E_: ventilation; VA: vertebral artery. Significant main effects are bolded. *P < 0.05 from baseline, ^†^P < 0.05 from lowlanders.

## Discussion

Our primary finding was iron status influences, albeit subtlety, global CBF that is inversely dependent on the severity and length-of-stay at high altitude. These data also highlight the reliance of iron and [Hb] on CBF responses to hypoxia, between lowlanders and highlanders. A secondary preliminary observation was that acute elevations in iron (and hence HIF downregulation) lead to a preferential elevation in posterior CBF in lowlanders at 4300 m. The following discussion outlines the implications and experimental considerations that underpin these findings.

### Cerebral blood flow, HIF expression, and iron status at high altitude

**Figure 3.**
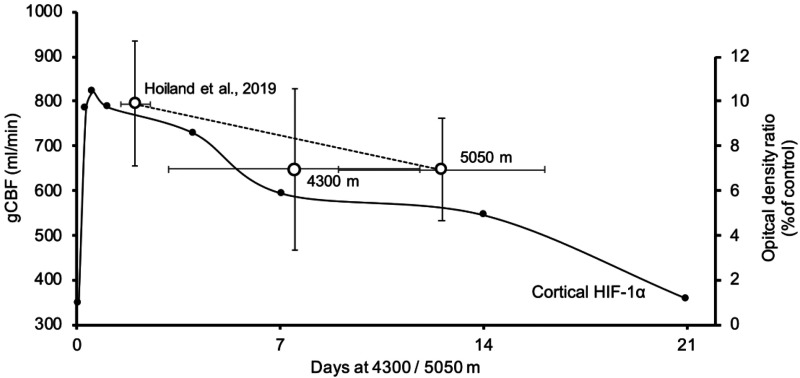
Summary of global CBF during prolonged stay at high altitude (5,050 m and 4,300 m refer to Studies 1 and 2, respectively). Cortical HIF-1α expression adapted from literature^11^ and CBF during early exposure to 5,050 in lowlanders adapted from literature.^3^ Since most of the lowlanders tested during Study 1 of the current study, were also included in the study by Hoiland and colleagues, the dashed line is included to illustrated the change in CBF across time.

During initial ascent and arrival to high altitude, it is well established that CBF increases in proportion to the reduction in arterial O_2_ content, in order to maintain cerebral O_2_ delivery. Over 1–2 weeks of acclimatization at a given altitude, CBF gradually normalizes to slightly above sea level values as erythropoiesis increases the O_2_ carry capacity of the blood and metabolic compensation occurs to partially correct the initial respiratory alkalosis [reviewed in:^50^]. As illustrated in Figure 3, it is noteworthy that the kinetics of cellular HIF expression also follows a similar trajectory during hypoxic exposure – in rodent models, HIF DNA binding activity reached 77% of maximal levels within one minute, and by 15 minutes HIF activity was detectable.^51^ In human endothelial cell culture models, HIF-1α and HIF-2α levels peaked after 4–6 hrs and 13 hours of hypoxic exposure, respectively.^52^ Interestingly, HIF-1α protein expression within the brain is more prevalent [versus HIF-2α^53^] and HIF-1α sensitivity to hypoxia is greater than other tissues^54^ – for example, an F_I_O_2_ of 0.18 was sufficient to induce HIF-1a protein expression in the brain, whereas F_I_O_2_ of 0.06 was needed for hepatic and renal tissue cells.^54^ In mouse cortical tissue, HIF-1α expression in the brain peaks at 6–12 hours and normalizes within ∼3 weeks.^11^

Iron – due to its constituent role in HIF stabilization, via prolyl hydroxylase activity^13^ – can influence the coordination of HIF-mediated responses designed to maintain O_2_ delivery.^55^ However, because iron deficiency commonly occurs with sojourn to high altitude,^16,17,55,56^ the responsiveness at which downstream HIF responses are stimulated might be influenced by the individuals’ prevailing iron status. This notion is based on our finding that iron infusion attenuated gCBF in lowlanders and Andeans at 4,300 m, but not in lowlanders and Sherpa at 5,050 m. To reconcile this discrepancy, the impacts of iron manipulation on the cerebral vasculature, between Study 1 & 2, should be interpreted within the context of degree and duration at altitude where the iron status of participants between Studies 1 & 2 were not identical. In Study 1 (5,050 m), 65% of lowlanders and 9% of Sherpa had ferritin levels of <15 ng · ml^−1^, while in Study 2 (4,300 m), only 14% of lowlanders and 4% of Andeans had ferritin levels <15 ng · ml^−1^.

While we would postulate that a greater prevalence of iron depletion at pre-infusion, would enhance the potency of iron infusion on altering cerebral vascular function, our findings that 1) iron infusion did not attenuate gCBF at 5,050 m, and 2) DFO did not markedly increase gCBF at 5,050 m. Together these data indicate that at least for the brain at this altitude, the characteristic rise in arterial O_2_ content^57^ plays a likely more potent role in CBF regulation since HIF expression would be expected to be declining over this timeframe.^11^

### Hemoglobin and serum iron

It is well established that [Hb] inversely dictates cerebral blood flow, via proportional changes to arterial oxygen content.^57,58^ However, tissue HIF activity also inversely coincide with arterial oxygen content,^59^ and iron modulates critical cofactors (e.g. prolyl-hydroxylases) important for HIF activity.^10,60^ At high altitude, the ubiquitous erythropoetic response disrupts iron tissue balance, which is reflected in an overall decline in iron levels. While Hb formation is certainly dependent upon available iron, changes in serum iron are earlier indicators of alternating demand/storage signals – and thus our findings of iron’s influence on ΔgCBF is notable. Ultimately, circulating iron levels and [Hb] are inextricably linked, but based on our correlational analyses, we are unable to tease out the independent influences of iron and Hb on CBF. Future studies are warranted, potentially in anemic and non-anemic volunteers who remain iron deplete or replete, to investigate this observation further.

### Exaggerated vertebral artery blood flow during hypoxia

Since the VA provides blood flow to the posterior region of the brain, including the brainstem – a key site of cardiorespiratory control – some studies have shown that posterior regions of the brain demonstrate a preferential blood flow distribution during hypoxia.^61–63^ Therefore, our finding of greater ΔQ_VA_ distribution (i.e. the elevation in VA flow from room air to exaggerated hypoxia) with iron in lowlanders at 4,300 m, may suggest that HIF activity, may aid in the regulation of flow to these highly homeostatic functioning regions of the brain.

### Role of high altitude ancestry

While high altitude Sherpa and Andeans display adaptive characteristics to hypoxia, including positive selection for HIF pathway candidate genes,^42,43^ there appears to be little evidence from the current study that iron manipulation differentially impacts cerebrovascular reactivity to hypoxia between partially acclimatized lowlanders and healthy highlanders. While this contrasts with our hypothesis, we acknowledge that we were only able to assess the acute (i.e., 4 hours) impact of HIF up-/down-regulation (via iron manipulation) on CBF. Since many of the downstream constituents of HIF are only evident after days-weeks (e.g. changes in cerebral microvascular density and hematocrit,^12^ neurovascular angiogenesis and pericyte proliferation^14^), the potential for cerebrovascular differences between high altitude residents and lowlanders to emerge over time warrants further investigation.

### Methodological considerations

While physiological assessments were consistent across both expeditions, it must be acknowledged that ascent profiles were not identical (especially in Study 1, where some Sherpa has ascended alongside lowlanders, and others did not – see section *Study 1 – Lowlanders and Sherpa at 5,050 m*). However, because iron status can vary markedly between individuals, and there were no differences between Sherpa ascending versus not-ascending (e.g., pre-infusion serum iron P = 0.134), we opted to include, rather than exclude the ascent Sherpa.

Sherpa (and Andeans) are typically smaller in height and weight, compared to westerners. Scaling gCBF (in ml/min) to brain mass (in ml/min/100g tissue) can be estimated using an allometrically scaling equation.^64^ A study by Hoiland and colleagues^3^ demonstrated brain mass of Sherpa to be ∼2–4% smaller, compared to lowlanders. While of interest, as discussed by the authors,^3^ the differences in global CBF were unlikely to be dependent solely on this notion, and instead more dependent upon a down-regulation of metabolic processes. Likewise, in a separate study, brain size (via T1 MRI imaging) between Han Chinese and lowlanders were not different.^65^

Globally, females typically have lower iron levels compared to males – a feature likely due to a combination of factors ranging from menstrual blood volume losses, reduced dietary intake/absorption and pregnancy.^66^ Similarly, females also demonstrate low iron levels at high altitude.^67^ Unfortunately, the ability to recruit more female participants in the current study was not possible. While the inclusion of females may make the sample population more heterogenous, we are unaware of any evidence of any measured or examined iron-related sex-differences in indigenous populations at altitude, include the Sherpa or Andeans. Ultimately, there exists a gap in our understanding of sex-differences at altitude, and as it pertains to iron metabolism – so further investigation, explicitly focused females and iron, is certainly warranted.

### Conclusion

In both healthy lowlanders and highlanders at high altitude, prevailing iron status appears to contribute to the variability in cerebral hypoxic reactivity. However, acute manipulation of iron status only minimally influences cerebral blood flow and function, that is potentially dependent on the severity and length of high altitude exposure. Given the variable and progressive depletion of iron stores at high altitude, broadening the scope of iron metabolic assessments to >24 hours post-infusion, may provide new insightful into potential relationships between iron stores and cerebral blood flow control during chronic hypoxia.

## Data Availability

The data that support the findings of this study are available from the authors upon reasonable request.
